# Transitivity performance, relational hierarchy knowledge and awareness: Results of an instructional framing manipulation

**DOI:** 10.1002/hipo.22163

**Published:** 2013-08-05

**Authors:** Dharshan Kumaran, Hans Ludwig

**Affiliations:** 1 Institute of Cognitive Neuroscience, University College London17 Queen Square, United Kingdom; 2 Department of Psychology, University of TorontoToronto, Canada

**Keywords:** hippocampus, transitive inference, generalization, relational memory

## Abstract

The transitive inference (TI) paradigm has been widely used to examine the role of the hippocampus in generalization. Here we consider a surprising feature of experimental findings in this task: the relatively poor transitivity performance and levels of hierarchy knowledge achieved by adult human subjects. We focused on the influence of the task instructions on participants’ subsequent performance—a single-word framing manipulation which either specified the relation between items as transitive (i.e., OLD-FRAME: choose which item is “older”) or left it ambiguous (i.e., NO-FRAME: choose which item is “correct”). We show a marked but highly specific effect of manipulating prior knowledge through instruction: transitivity performance and levels of relational hierarchy knowledge were enhanced, but premise performance unchanged. Further, we show that hierarchy recall accuracy, but not conventional awareness scores, was a significant predictor of inferential performance across the entire group of participants. The current study has four main implications: first, our findings establish the importance of the task instructions, and prior knowledge, in the TI paradigm—suggesting that they influence the size of the overall hypothesis space (e.g., to favor a linear hierarchical structure over other possibilities in the OLD-FRAME). Second, the dissociable effects of the instructional frame on premise and inference performance provide evidence for the operation of distinct underlying mechanisms (i.e., an associative mechanism vs. relational hierarchy knowledge). Third, our findings suggest that a detailed measurement of hierarchy recall accuracy may be a more sensitive index of relational hierarchy knowledge, than conventional awareness score—and should be used in future studies investigating links between awareness and inferential performance. Finally, our study motivates an experimental setting that ensures robust hierarchy learning across participants—therefore facilitating study of the neural mechanisms underlying the learning and representation of linear hierarchies.

## INTRODUCTION

The transitive inference (TI) paradigm has been extensively used across species to investigate the cognitive mechanisms supporting generalization—a term we use here to describe the flexible expression of prior learning in novel situations (e.g., new associative combinations of familiar stimuli), through the exploitation of structure present within a set of related episodes (e.g., Kumaran, 2012; Kumaran and McClelland, 2012; Zeithamova and Zeithamova et al. 2012)—and the specific contribution of the hippocampus (Bryant and Trabasso, 1971; McGonigle and Chalmers, 1977; Rapp et al., 1996; Dusek and Eichenbaum, 1997; Delius and Siemann, 1998; Greene et al., 2001; Frank et al., 2003,2005; Heckers et al., 2004; Titone et al., 2004; Smith and Squire, 2005; Moses et al., 2006, b; Kumaran et al., 2012; Zeithamova et al., 2012). Indeed, recent work has highlighted the theoretical significance of an important role for the hippocampus in generalization, which offers a substantial challenge to prevailing computational accounts of this neural structure as an episodic memory device which by its nature tends to emphasize the distinct features of events, at the expense of their commonalities (e.g., Kumaran, 2012; Kumaran and McClelland, 2012; Zeithamova et al).

Two puzzling questions remain, however; first, it has not been clear why the ability of adult human subjects to perform transitivity judgments in contemporary versions of the paradigm tends to be highly variable across participants and between studies (e.g., ∼60% in Frank et al., 2005 vs. ∼90% in Greene et al. 2001), and often relatively poor. Indeed, the variable performance of adult subjects stands in contrast to an early study which reported high levels of transitivity performance (∼80–90%) in young children of 4–6 yrs of age (Bryant and Trabasso, 1971). Second, and related to the issue of variable transitivity performance, is the question of why only a minority of subjects typically develop relational (or declarative) knowledge of the hierarchy, as indexed by the accuracy with which participants are able to recall the order of items in hierarchy (e.g., Smith and Squire, 2005)—e.g., 8 out of 65 participants in the experiment by Frank et al., 2005.

Here we investigate why subjects tend to show relatively poor transitivity performance and often fail to develop hierarchy knowledge, in versions of the TI paradigm that could be considered the standard in the contemporary literature (Greene et al., 2001; Heckers et al., 2004; Frank et al., 2005; Smith and Squire, 2005; Moses et al., 2006; Ellenbogen et al., 2007; Moses et al., 2010b; Zeithamova et al., 2012). In particular, we focused our attention on the potential influence of the task instructions received by participants. Our hypothesis was that the strikingly proficient levels of transitivity performance (i.e., >80%) shown by the very young subjects (i.e., 4–6 yrs old) in the original paradigm used by Bryant and Trabasso (1971) could in part have related to the inherently transitive nature of the relation between items in that setting (i.e., selecting the “longer” of a pair of rods—where actual rod length was hidden from view). Accordingly, our intuition was that the relatively poor and variable levels of transitivity performance exhibited by participants in the “standard” version of the paradigm (reviewed in Zeithamova et al., 2012) might be accounted for by instructions that leave the nature of the relation ambiguous (i.e., “choose which item is correct”). As such, participants in recent TI experiments may equally well have assumed on the basis of training trial experiences that the relationship between items is contextual (i.e., choose A in the presence of AB, but B in the presence of BC; see Greene et al., 2001<AQ2> for a related argument), rather than transitive in nature—and consequently performed relatively poorly as a group during transitivity trials (e.g., B-D).

To test the hypothesis that task instructions are an important factor in influencing participants' subsequent level of transitivity performance and hierarchy knowledge, we conducted a between-subjects experiment. Participants in each experimental group were treated identically, with the exception of the initial task instructions received, which differed by one critical word—as such the relation between items was either left ambiguous, or specified to be transitive in nature (see Methods). In contrast, previous studies either assessed the effect of manipulating the overall instructions received by participants (Greene et al., 2001), or providing prior experience with a semantically meaningful hierarchy composed of playing cards (i.e., 9–10-J-Q-K-A: Moses et al., 2010a)—rather than specifically focusing on the way in which item relations are specified (see Discussion).

Participants in the NO-FRAME group were instructed to choose the stimulus, depicting a galaxy, which they thought was “correct” during training trials, and test trials. As such, the instructions received by participants in the NO-FRAME group were designed to be analogous to those used in what we refer to as the standard TI paradigm (reviewed in Zeithamova et al., 2012). In contrast, participants in the OLD-FRAME group were instructed to choose the stimulus (i.e., galaxy) they thought was “older” during training and test trials—following the “classical” version of the TI paradigm developed by Bryant and Trabasso (Bryant and Trabasso, 1971). Our hypothesis was that participants in the OLD-FRAME group would show significantly better inference performance, through having the relation being items defined (cf., left ambiguous—in the NO-FRAME group) at the point of instruction. Further, we aimed to show, through a direct test of the accuracy of hierarchy recall, that this inferential performance was underpinned by relational knowledge (Eichenbaum, 2004; Smith and Squire, 2005) of the hierarchy (cf., other mechanisms: see Moses et al. 2006 and Zeithamova et al., 2012, for review).

## MATERIALS AND METHODS

### Participants

Thirty healthy individuals participated in this experiment (age range 18–35; 14 female). All participants gave informed written consent to participation in accordance with the local research ethics committee. Each group (NO-FRAME, OLD-FRAME) comprised 15 participants. There was no significant differences between subject groups in terms of age, years of higher education, or performance on an abbreviated form (i.e., subset) of the Raven's progressive matrices (all *P*s >0.1).

Participants in each experimental group were treated identically, with the exception of the initial task instructions received, which differed through one critical word. Participants in the NO-FRAME group were instructed to choose the galaxy they thought was “correct” during training trials and test trials. In contrast, participants in the OLD-FRAME group were instructed to choose the galaxy they thought was “older” during training and test trials.

### Stimuli

Pictures of galaxies were obtained from various sites on the Internet (including http://hubblesite.org/gallery/album/nebula). The galaxy hierarchy consisted of eight items (i.e., A-B-C-D-E-F-G-H, where A is the highest ranking item and H the lowest ranking; see Appendix for example stimuli, though color stimuli were used in the experiment). The allocation of individual pictures to position in the hierarchy was randomized across the group of participants. Stimuli were presented using Cogent Graphics toolbox (http://www.vislab.ucl.ac.uk/cogent_graphics.php) operating in a MATLAB 7 environment.

### Experimental Design

During a training trial, adjacent items in the hierarchy were presented on either side of the screen (i.e., seven premise pairs: e.g., A vs. B, B vs. C, C vs. D, D vs. E, E vs. F, F vs. G, G vs. H). The left-right position of an item on the screen was randomized across trials. Participants had 5 seconds in which to choose, via button press (i.e., left or right, index or middle finger of right hand, respectively), the item which they thought was “correct” (NO-FRAME condition) or “older” (OLD-FRAME condition). After participant's response, a feedback screen appeared: this consisted of a green square border that indicated the participant's choice together with either “+20 points” or “−20 points”, for a correct or incorrect response, respectively. A fixation cross preceded the onset of the next trial.

Test trials involved the presentation of the seven premise pairs as well as nine inference pairs (i.e., pairs of nonadjacent items in the hierarchy: B vs. D, B vs. E, B vs. F, C vs. E, C vs. F, C vs. G, D vs. F, D vs. G, E vs. G). As in training trials, participants had 5 seconds in which to choose, via button press (i.e., left or right), the galaxy which they thought was “correct” (NO-FRAME condition) or was “older” (OLD-FRAME condition). Importantly, feedback was not presented during test trials, although participants were instructed that their choices would still count toward their final payout. Instead, after participant's response, a screen appeared that required participants to rate (on a scale of 1–3) their confidence in their decision: participants were carefully instructed to enter a “1” response if they were guessing entirely, a “2” response if they “had some idea but were not sure” about their choice, and to reserve a “3” response until they were “more than 90% certain” that their choice was the correct one. Participants were told that although their confidence responses would not count toward their final payout, they should still answer as accurately as possible. Note that although subjective confidence responses were recorded in this experiment, here we restrict our analyses to the binary choice data.

The remuneration received by participants was determined directly from the number of correct responses during training and test trials, in addition to a basic minimum for participation in the experiment.

Participants completed two experimental sessions (with a 1-minute break in between), comprising six blocks each. Each block consisted of a miniblock of training trials followed by a miniblock of test trials. Each training trial miniblock consisted of 21 trials in total made up from three repetitions of each of the seven premise pair types (e.g., A vs. B: see above) presented in pseudorandom order. Each test trial miniblock consisted of 16 trials in total made up from one repetition of each of the seven premise pairs (e.g., A vs. B), and one repetition of each of the nine inference pairs (e.g., B vs. D), presented in pseudorandom order. The start of each miniblock was preceded with the relevant instruction (i.e., “Get ready for Training trials”, “Get ready for Test trials”). Following the end of each miniblock, a screen showing the percentage of correct responses achieved was displayed.

### Debriefing Protocol and Scoring of Hierarchy Recall Test

Following the completion of the experiment, participants were carefully debriefed so as to evaluate the presence and nature of explicit knowledge concerning the task structure. A debriefing questionnaire (total score = 12; see Appendix) that was based closely on that employed by Moses et al. (2006) (also see Greene et al., 2001) was administered to participants to assess their explicit awareness of the task structure (i.e., including awareness that item relations were transitive). This questionnaire included among other questions, a hierarchy recall test in which participants were asked to explicitly reconstruct the order of items in the hierarchy.

Participants' performance on the hierarchy recall test was scored in two ways: (1) on a two-point scale, as in Moses et al., 2006<AQ2>, which counted toward the 12-point awareness score; (2) using a scoring procedure that penalizes incorrect positioning of an item according to its deviation from the item's true position. Specifically, the summed deviation of a participant's stated position of each item from its true position (e.g., if the top ranked item was placed in sixth position, this would be scored as a deviation of 5) was calculated.

### Behavioral Analyses

Behavioral analyses were conducted using SPSS version 19 using standard procedures. The analyses presented here focus on the binary choice data obtained during training and test trials: the test trial confidence data are not considered for the present purposes. Performance accuracy and reaction time were analyzed for both training blocks and test blocks, using repeated measures mixed factor analyses of variance (ANOVA). Mauchly's test was used to evaluate whether the assumption of sphericity had been violated, and degrees of freedom were corrected using Greenhouse-Geisser estimates of sphericity when appropriate.

## RESULTS

### Performance on Training Trials

**Figure 1:**
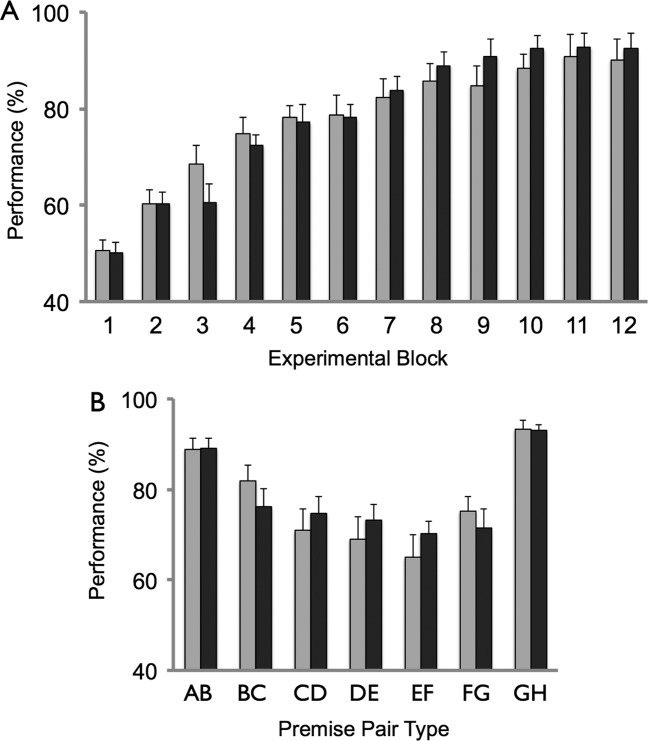
Performance during training trials. (A) Performance on training trials across the 12 blocks of the experiment. (B) Performance as a function of premise pair type, averaged across all experimental blocks. NO-FRAME group shown in light gray, and OLD-FRAME group shown in dark grey. Error bars denote SEM.

We first considered the performance (% correct and reaction time (RT)) of both groups of participants over the 12 blocks of training trials. During the last block of training trials, the NO-FRAME group averaged 90.2% correct responses (SD 16.3), and the OLD-FRAME group averaged 92.4% correct responses (SD 9.0)(see Fig. 1A). Data were analyzed using a mixed-design ANOVA with one within-subject factor of block (12 levels) and one between-subject factor (group: NO-FRAME, OLD-FRAME). In terms of % correct responses, main effect of block (*F*(3.9, 108.8) = 56.0, *P* < 0.001) was significant, with a significant linear effect (*F*(1, 28) = 118.7, *P* < 0.001). Main effect of group and interaction of block and group were not significant (*P* > 0.2). For RT: main effect of block (*F*(4.1, 115.5) = 7.18, *P* < 0.001) was significant, with a significant linear effect (*F*(1, 28)=18.2, *P* < 0.001). Main effect of group and interaction of block and group were not significant (*P* > 0.2).

We next considered the performance of participants on training trials, as a function of premise pair (see Fig. 1B). Data were collapsed across all training trial blocks and analyzed using a mixed-design ANOVA with one within subject factor of premise pair type (seven levels: A vs. B, B vs. C…, G vs. H), and one between-subject factor of group (NO-FRAME, OLD-FRAME). For % correct responses: main effect of premise pair type (*F*(6, 168) = 20.7, *P* < 0.001) was significant, with a significant quadratic effect (*F*(1, 28) = 116.3, *P* < 0.001). Main effect of group and interaction of premise pair type and group were not significant (*P* > 0.2). For RT: we observed a main effect of premise pair type (*F*(4.2, 117.9) = 56.8, *P* < 0.001). No effect of group, or group by premise pair interaction was observed (*P* > 0.2).

As a follow-up test, we explored the main effect of premise pair type further in a mixed-design ANOVA where premise pairs were grouped into outer pairs (A vs. B, B vs. C, F vs. G, and G vs. H) and inner pairs (C vs,D, D vs. E, E vs. F): a main effect of pair type was found (*F*(1, 28) = 61.9, *P* < 0.001) reflecting superior performance on trials involving outer pairs. Indeed, performance on outer pairs was significantly better than on inner pairs when the outermost premise pairs were excluded from this analysis (i.e., outer pairs consisted only of B vs. C, F vs. G: *F*(1,28) = 64.5, *P* < 0.001). For RT: a main effect of pair type was also found (*F*(1, 28) = 81.1, *P* < 0.001) reflecting shorter reaction times on trials involving outer pairs.

We further observed that performance (% correct) was superior during GH trials, as compared to AB trials as evidenced by the results of a mixed-design ANOVA (factors: premise pair type, group: (*F*(1, 28) = 9.4 *P* < 0.01)) and a paired-sample *t*-test (GH > AB: *t*(29) = 3.1, two-tailed test *P* < 0.01). Reaction times were also significantly faster during GH trials, as compared to AB trials: *t*(29) = 2.1, two-tailed test *P* < 0.05).

These results demonstrate that participants improved their performance across training trial blocks, in terms of % correct responses and speeded reaction times, with no difference observed between NO-FRAME and OLD-FRAME groups. Further, the profile of performance observed (e.g., superior performance in outer premise pairs vs. inner pairs) was similar in both subject groups, and consistent with the operation of associative learning mechanisms (Delius and Siemann, 1998; Frank et al., 2003; Frank et al., 2005; Moses et al., 2006; Zeithamova et al., 2012).

### Performance on Test Trials

**Figure 2:**
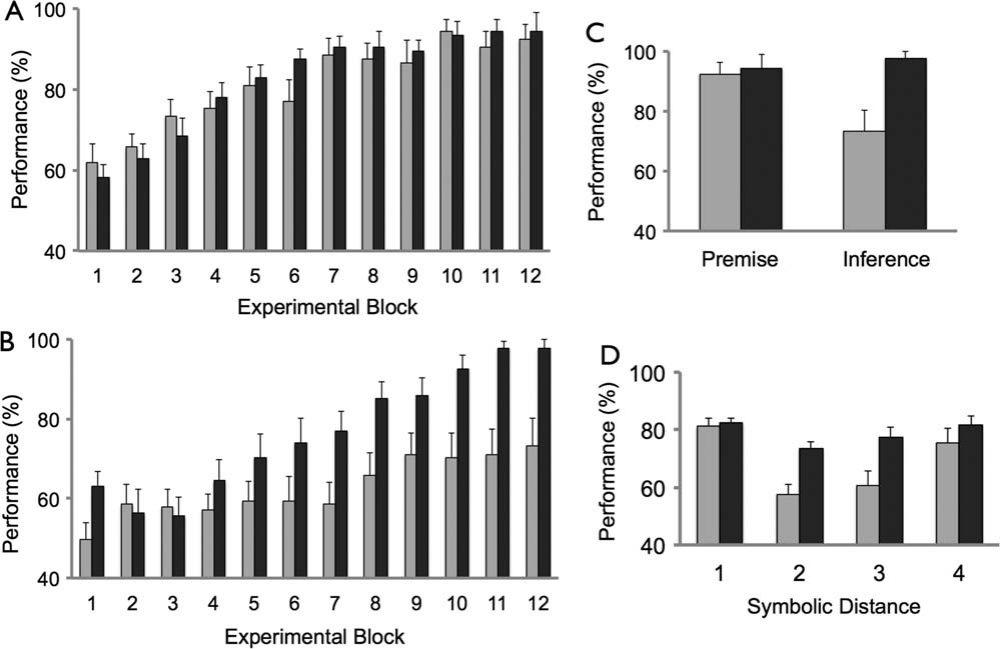
Performance during test trials. (A) Performance on test trials involving premise pairs (e.g., A vs. B) across the 12 blocks of the experiment. (B) Performance on test trials involving inference pairs (e.g., B vs. D) across the 12 blocks of the experiment. (C) Performance on test trials (i.e., premise and inference pairs) during the last block. (D) Performance on test trials as a function of symbolic distance (i.e., premise pair = 1, B vs. D inference pair = 2 etc.) NO-FRAME group shown in light gray, and OLD-FRAME group shown in dark grey in all plots. Error bars denote SEM.

Although confidence responses were also obtained in this experiment, here we focus our analysis on the binary choice data (see Methods). The average performance of participants in the NO-FRAME group on premise pairs and inference pairs across all test trial blocks was 81.1% (SD 10.5) and 62.6% (SD 14.4), respectively. The average performance of participants in the OLD-FRAME group on premise pairs and inference pairs was 82.5% (SD 5.9) and 76.7% (SD 10.1), respectively (see Fig. 2A,B). During the last test trial block (i.e., block 12), the average performance of participants in the NO-FRAME group on premise pairs and inference pairs was 92.4% (SD 15.3) and 73.3% (SD 26.8), respectively. The performance of participants in the OLD-FRAME group on premise pairs and inference pairs during the last test trial block was 94.3% (SD 18.6) and 97.8% (SD 8.6), respectively (see Fig. 2C).

Data were analyzed using a mixed-design ANOVA with two within-subject factors of trial type (premise, inference) and block, and one between-subject factor (group: NO-FRAME, OLD-FRAME). For % correct responses: main effects of trial type (*F*(1, 28) = 29.2, *P* < 0.001), block (*F*(5.1, 143.8) = 29.8, *P* < 0.001), and group (*F*(1, 28) = 6.0, *P* < 0.05) were significant. There were significant interactions between trial type and group (*F*(1, 28) = 7.9, *P* < 0.01), and trial type and block (*F*(6.0, 169.1) = 2.3, *P* < 0.05). A borderline significant result was observed for the interaction between block and group (*F*(5.1, 143.8) = 2.2, *P* = 0.06). For RT: main effects of trial type (*F*(1, 28) = 38.3, *P* < 0.001), block (*F*(5.0, 139.4) = 17.8, *P* < 0.001), and block by group interaction (*F*(5.0, 139.4) = 2.6, *P* < 0.05) were significant. The main effect of group was not significant (*P* > 0.1). A follow-up paired sample *t*-test showed that premise pair RT was significantly faster (mean 1.8 seconds, SD 0.3) than inference pair RT (mean 2.0 seconds, SD 0.3: *t*(29) = 5.7, *P* < 0.001).

A further ANOVA confirmed that the significant main effect of group (% correct responses), and interaction between group and trial type, was driven by superior performance of the OLD-FRAME group compared to the NO-FRAME group on inference pairs. Specifically, an ANOVA focusing on inference pair performance (i.e., factors: block, group) revealed a significant main effect of group (*F*(1, 28) = 9.6, *P* < 0.01), as well as a main effect of block (*F*(5.4, 149.8) = 14.5, *P* < 0.001). Of note there was also a significant block × group interaction (*F*(5.4, 149.8) = 2.6, *P* < 0.05). This was shown in a follow-up ANOVA to be due to significantly superior inference pair performance in the OLD-FRAME group (cf., NO-FRAME) in the second half of the experiment (main effect of group: *F*(1, 28) = 14.4, *P* < 0.001), but not in the first half (*P* > 0.2). In contrast, an ANOVA focusing on premise pair performance (i.e., factors: block, group) revealed no significant main effect of group or block by group interaction (both *P*s > 0.2), although a significant main effect of block was present (*F*(5.7, 160.6) = 24.1, *P* < 0.001).

We next examined how performance on inference pairs varied as a function of the distance between the position of items on the hierarchy (i.e., symbolic distance; e.g., equal to 2 for a B vs. D trial; see Fig. 2D). Relating to % correct responses: an ANOVA with factors distance (2, 3, 4) and group showed a significant main effect of distance: *F*(2, 27) =15.9, *P* < 0.001) as well as a significant main effect of group (*F*(1, 28) = 7.6, *P* < 0.01). Although there was a borderline significant distance × group interaction: *F*(2, 27) = 3.1, *P* = 0.06), we found a main effect of distance when each subject group was analyzed separately: NO-FRAME ((*F*(2, 28) = 11.6, *P* < 0.001) and OLD-FRAME ((*F*(2, 28) = 4.7, *P* < 0.05)), with both effects linear in nature (both *P*s <0.01). No significant effects were seen in relation to RT, either in terms of a main effect of distance or a distance by group interaction (both *P*s >0.1).

Follow-up one-way ANOVAs revealed that these results were driven by a superior performance (% correct responses) of the OLD-FRAME group (cf., NO-FRAME) in distance 2 (e.g., B vs. D) and distance 3 (e.g., B vs. E) inference pairs: *F*(1, 28) = 14.9, P < 0.001 and *F*(1, 28) = 7.8, *P* = 0.01, respectively. In contrast, no difference was found between performance of the two groups in distance 4 inference pairs: *P* > 0.2 (see Fig. 2D).

### Awareness Scores and Hierarchy Recall Accuracy

The average of the NO-FRAME group on the “awareness” post-experimental questionnaire test (maximum score achievable = 12 points) was 6.9 (SD 2.1)—that of the OLD-FRAME group was 9.5 (SD 2.7). These scores were significantly different: one-way ANOVA *F*(1, 28) = 8.3, *P* < 0.01.

**Figure 3:**
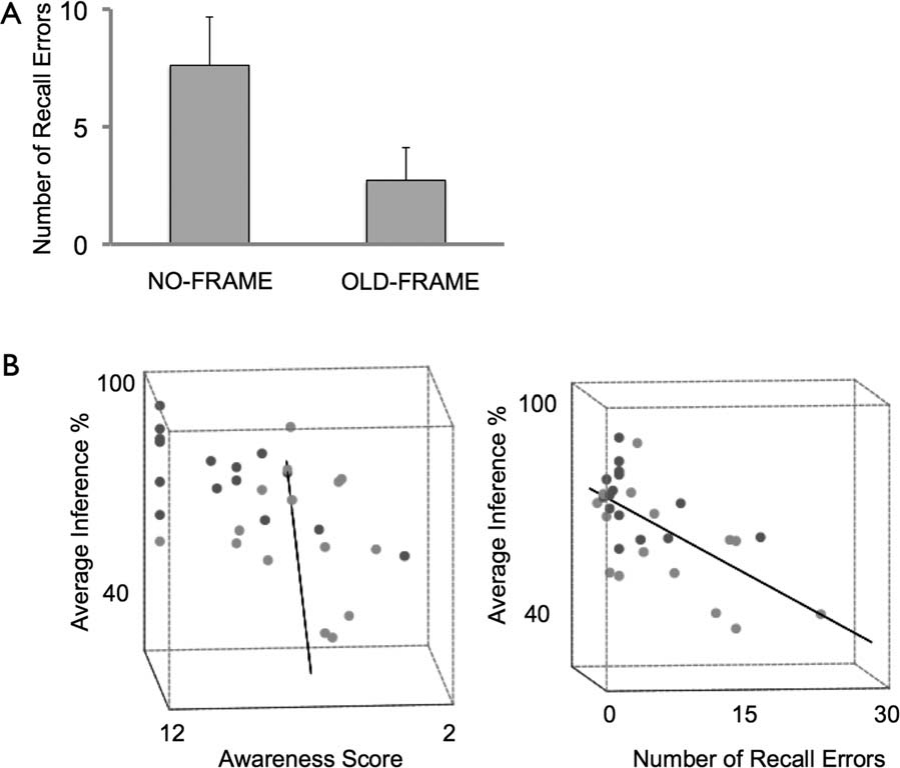
Performance on hierarchy recall test. (A) Average number of recall errors for each subject group. Error bars denote SEM. Note chance performance (calculated by monte carlo simulation) is 20.5 (SD 5) (B) 3D Scatterplot with regression line fitted, and two views illustrated: Lefthand plot shows absence of a significant influence of awareness score on inference performance on test trials (i.e., averaged across 12 blocks). Righthand plot shows significant linear correlation between number of hierarchy recall errors made by a given subject, and their performance on inference test trials (i.e., averaged across all 12 blocks).

Considering participants performance on the hierarchy recall test specifically: 5 out of 15 participants in the NO-FRAME were able to perfectly recall the hierarchy, as compared to 11 out of 15 participants in the OLD-FRAME group: these values were significantly different from each other (Pearson's chi-squared: *P* < 0.05). The average number of errors made on the hierarchy recall test (see Methods for details of calculation of deviation of participant's reconstructed hierarchy from actual hierarchy) for the NO-FRAME and OLD-FRAME groups were 7.6 (SD 8.1) and 2.7 (SD 5.6), respectively (see Fig. 3A). There was a borderline significant effect of group on the number of hierarchy recall errors made: *F*(1, 28) = 3.8, *P* = 0.06.

We next asked whether participants' score on the awareness questionnaire, and their ability to accurately recall the hierarchy order, correlated with their performance on inference pairs during test trials. When both groups were considered together, there was a significant correlation between performance on inference test pairs (i.e., averaged across all 12 blocks) and number of hierarchy recall errors (*r* = −0.67, *P* < 0.001)—and also with awareness score (*r* = 0.46, *P* < 0.05). Considering each group separately, in the NO-FRAME group, there was a significant correlation between inferential performance (averaged across all pair types in the entire experiment) and the accuracy of hierarchy recall (*r* = 0.63, *P* < 0.01), but not awareness score (*r* = 0.0, *P* > 0.2). Note that the correlation of hierarchy recall accuracy with inferential performance was also significant when only the last block of test trials was considered (*r* = 0.80, *P* < 0.001)—as before, no correlation with awareness score was observed (*P* > 0.1). In the OLD-FRAME group, both debriefing measures showed a significant correlation with inferential performance (awareness score *r* = 0.64 *P* < 0.01, hierarchy recall accuracy *r* = 0.58, *P* < 0.05).

Finally, we performed a multiple regression analysis where the relationship between inferential performance (i.e., averaged across blocks) and the two debriefing measures (i.e., awareness score, number of hierarchy recall errors) was assessed, across the whole group of 30 participants. We found a significant overall effect (ANOVA: *F*(2, 27) = 11.1, *P* < 0.001) that was related to a significant effect of hierarchy recall errors (beta = −0.64, *P* < 0.001: see Fig. 3B) but not awareness score (beta = 0.04, *P* > 0.8). A similar result was also observed when inferential performance was restricted to the last test trial block (ANOVA: *F*(2, 27) = 23.6, *P* < 0.001): with a significant effect of hierarchy recall errors (beta = −0.78, *P* < 0.001) but not awareness score (beta = 0.03, *P* > 0.8). Taken together, these results suggest that high levels of relational hierarchy knowledge, but not high levels of awareness, were predictive of successful inferential performance.

## DISCUSSION

In the current study, we examined the influence on performance in the TI paradigm of manipulating prior knowledge at the point of instruction—through the change of a critical word from one that specified the relation between items as transitive (“older”) to one that left it ambiguous (“correct”). We report three key findings: first, our instructional manipulation had a highly specific effect on inferential performance, whilst leaving premise pair performance entirely unaffected. Secondly, we show that the OLD-FRAME ensured the development of robust relational hierarchy knowledge across the subject group—to a degree that was significantly greater than that achieved by the NO-FRAME group. Finally, we show that hierarchy recall accuracy, but not awareness scores, was a significant predictor of inferential performance across the entire group of participants—suggesting that this measure may be a more sensitive index of relational hierarchy knowledge. Taken together, our findings provide insights into the relatively poor performance of participants in contemporary TI experiments, motivate a version of the TI paradigm that ensures robust hierarchy learning, and highlight the critical importance of distinguishing between measures of hierarchy knowledge, and more general measures of awareness.

It is important to consider our experimental paradigm and findings in relation to two previous studies which share similar aims. We first summarize their principal findings: in the study by Greene et al. (2001), participants who were given explicit instructions revealing the task structure—concerning the transitivity of the item relation, the number of items in the hierarchy, and the structure of premise pair presentation (i.e., involving adjacent items in hierarchy) (see the Appendix of this paper for details)—performed more successfully on premise pairs and inference pairs, as compared to (“uninformed”) participants who were merely instructed to choose the “correct” item. The study by Moses et al. (2010a) observed a significant effect on both premise and inference pair performance using a very different form of between-subjects manipulation involving training: here, participants that had previously been exposed to and solved a TI task involving a semantically familiar hierarchy (i.e., a playing card hierarchy: 9–10-J-Q-K-A) performed better in a subsequent experimental session involving a hierarchy composed of abstract shapes, as compared to participants who had experienced only abstract stimuli during training.

Our findings, therefore, converge with previous work in demonstrating that the relatively poor performance of healthy young participants on contemporary versions of the TI task can be markedly improved through manipulating the amount of prior knowledge—either through instruction (Greene et al., 2001) or through previous training (Moses et al., 2010a; also see Kumaran, in press). A key difference between our study and previous work, however, is the selectivity of the between-subjects manipulation: in our experiment the two participant groups were treated equivalently, with the exception of a single word change at instruction (i.e., “correct” or “older”). In contrast, the two participants groups in these previous studies differed in several respects; i.e., not only in terms of information received concerning the nature of item relation (as in our study) but also in terms of their knowledge of other aspects of the task (e.g., number of items in the hierarchy, premise pair schedule, analogical equivalence with a hierarchy of playing cards). Importantly, therefore, our findings establish that merely manipulating prior knowledge of the nature of the item relation has a marked influence on transitivity behavior, whereas previous studies leave open the question of whether it is application of a semantically familiar schema (Moses et al., 1977), or knowledge of other aspects of the underlying task structure (Greene et al., 2001) that is critical. Indeed, a recent study provides evidence that these other kinds of information—e.g., knowledge of the structure of premise pair presentation acquired through training—may also facilitate new hierarchy learning (Kumaran, In Press).

The current findings—in identifying a critical source of information (i.e., item relation definition) that facilitates performance in the TI task—point toward a potential mechanism that may mediate the effects of instruction on transitivity performance. Specifically, instructing participants to choose the “older” item likely increased the prior over a linear hierarchical structure—a structural form intimately related to the concept of transitivity—in effect narrowing the hypothesis space through the activation of an “overhypothesis” (e.g., Kemp et al., 2007; Tenenbaum et al., 2011). Along these lines, the relatively poor performance of participants in recent TI experiments (reviewed in Zeithamova et al., 2012), and the NO-FRAME condition, may have been due to the relatively unconstrained nature of the resulting hypothesis space: participants may have equally well assumed on the basis of training trial experiences that the relationship between items was contextual (i.e., choose A in the presence of AB, but B in the presence of BC), rather than hierarchical (i.e., transitive) in nature. Indeed, one could speculate that in ethological settings (Cheney and Seyfarth, 1990; Cheney and Seyfarth, 2007), primates are only able to efficiently acquire knowledge about their social hierarchy from dyadic interactions (cf., training trials) because the relationship (i.e., dominance) between the individuals in a dyadic interaction is inherently specified to be transitive in nature.

Interestingly, our experimental manipulation was found to have a specific influence on test trial, but not training trial, performance—in contrast to the improvement of both training and test trial performance observed by Greene et al. (2001) and Moses et al. (2010a). Notably, the performance of both subject groups in our experiment on premise pairs (i.e., during training and test trials) was indistinguishable, both in terms of overall % correct responses and overall RT, and also the profile of performance observed across different pairs, consistent with the operation of associative learning mechanisms (i.e., U-shaped: see Fig. 1B)(e.g., Zeithamova and Zeithamova et al. 2012).

Whilst the important methodological differences between studies makes it difficult to establish why our experimental manipulation alone produced a specific effect on transitivity performance and hierarchy knowledge, one possibility is that manipulations in these other studies resulted in more general changes in task strategy and hence more widespread effects on performance. Regardless of the exact mechanism, the experimental dissociation observed—between premise pair performance on the one hand, and transitivity performance and relational hierarchy knowledge on the other—provides evidence that relational knowledge of the hierarchy does not necessarily emerge once memory of the premise pairs reaches a high level, a possibility that has been left open based on prior work in humans, which has tended to observe that these two indices of performance tend to rise and fall in parallel (e.g., Smith and Squire, 2005; Moses et al., 2006). As such, the current data point toward dissociable underlying mechanisms (i.e., associative vs. relational, respectively), with only the latter (i.e., relational) under the influence of our instructional framing manipulation—and accords with previous studies showing that inferential performance, but not premise performance, is disrupted by hippocampal lesions in non-humans (Dusek and Eichenbaum, 1997; Buckmaster et al., 2004).

It is interesting to consider the neural mechanisms mediating the enhancement of hierarchy learning by our instructional framing manipulation. Although future studies are needed to explore this question, one possibility is that this effect depends on interactions between the hippocampus and prefrontal cortex (PFC). Whilst substantial work has focused on the critical role of the hippocampus (e.g., see for review, Eichenbaum, 2004; Zeithamova and Zeithamova et al. 2012), the PFC is also thought to make an important contribution to transitivity behavior based on lesion evidence (DeVito et al., 2010; Koscik and Tranel, 2012) and neuroimaging studies in humans (Acuna et al., 2002; Wendelken and Bunge, 2009; Heckers et al., 2004; Kumaran et al., 2012)—although notably different subregions of PFC (e.g., ventromedial PFC in Kumaran et al., 2012; dorsolateral PFC in Acuna et al., 2002; rostrolateral PFC in Wendelken and Bunge, 2009) have been highlighted in these studies. More generally, the PFC is viewed to support successful performance in “problem solving” tasks—of which the transitive inference task can be considered one example—through rule learning at multiple levels of abstraction and relational complexity (e.g., Koechlin and Summerfield, 2007; Badre, 2008; Badre et al., 2010). As such it is tempting to speculate that our instructional manipulation acts to constrain the hypothesis space through the shaping of higher-order representations of the overall task structure in PFC, which ultimately influence the formation of memory representations of the linear hierarchy in the hippocampus.

It is worth considering how participants' performance on test trials varied as a function of the symbolic distance between items in the hierarchy. Both groups performed significantly better when transitive inferences involved larger symbolic distances, showing linearly increasing performance over the range examined (i.e., 2–4). Interestingly, participants in the OLD-Frame group performed significantly better than the NO-Frame group at transitive inferences involving a smaller symbolic distance (i.e., 2 or 3), but there was no significant difference between the groups at a larger symbolic distance (i.e., 4). Whilst mechanisms based on both associative learning and hierarchy knowledge are known to produce symbolic distance effects of this nature (Breslow, 1981; Delius and Siemann, 1998; Moses et al., 2006; Zeithamova et al., 2012), our data point to the conclusion that test trial performance in both groups was driven primarily by relational hierarchy knowledge (Cohen and Eichenbaum, 1993; Eichenbaum, 2004; Smith and Squire, 2005) for two reasons: first, associative mechanisms are typically viewed to make significant contributions to test trial performance in the case of shorter hierarchies than used in this experiment (i.e., five or six items, as opposed to eight) (Frank et al., 2003). Second, we found a significant correlation in both groups between test trial performance and participants' level of hierarchy knowledge, indexed by the hierarchy recall test. As such, our data are consistent with the conclusion that test trial performance in the NO-Frame group reflected the use of hierarchy knowledge, which was sufficient to mediate comparable levels of performance (cf., the OLD-Frame group) at large, but not smaller, symbolic distances.

Our study also has implications for a contentious debate surrounding the relationship between the ability to make transitive inferences during inference trials and measures of “awareness” (Greene et al., 2001; Frank et al., 2005; Smith and Squire, 2005)—an issue that has wider relevance to the question of whether the hippocampus contributes not only to “explicit”, but also “implicit”, memory (e.g., Greene, 2007). A controversial finding from previous studies is that healthy participants may perform successfully (i.e., above chance levels) on transitivity test trials, yet not exhibit conscious awareness (i.e., explicit knowledge) of the underlying hierarchy—measured typically by questionnaires administered at the point of debriefing (e.g., Greene et al., 2001; Frank et al., 2005). Our findings, however, suggest that such questionnaires may not be sufficiently sensitive—a critical consideration in assessing the validity of awareness tests (Shanks and St John, 1994)—in detecting the presence of explicit hierarchy knowledge. Specifically, our findings demonstrate that there was a robust correlation between inferential performance in the NO-Frame group and our continuous measure of hierarchy recall accuracy—which can be considered a gold standard for indexing relational knowledge of the hierarchy (e.g., see Smith and Squire, 2005; Kumaran et al., 2012)—even when no such correlation was evident between conventional questionnaire scores. Indeed we show, in a multiple regression analysis across the whole group of 30 participants, that high levels of relational hierarchy knowledge, but not high awareness scores, were strongly predictive of inferential performance.

Our findings, therefore, suggest the need for caution when interpreting previous reports that successful transitivity performance is possible in the absence of explicit knowledge of the underlying hierarchy (e.g., Greene et al., 2001; Frank et al., 2005). Whilst we believe that future studies are needed to definitively establish whether above-chance levels of inferential performance are possible in the absence of relational hierarchy knowledge—where this is indexed by a continuous hierarchy recall measure—we do acknowledge that this may well be the case. Indeed, under a different experimental conditions (e.g., using other training regimes), it is conceivable that successful transitive inference performance may be putatively supported by “implicit” relational hierarchy representations (e.g., Greene, 2007), or alternative associative learning mechanisms (e.g., Frank et al., 2004), which may be inaccessible to conscious awareness—and therefore uncorrelated with both awareness scores, and performance on a hierarchy recall test.

Linear hierarchies can be considered a prototypical form of knowledge structure that have a wide-ranging influence across cognitive domains (Kemp and Tenenbaum, 2008). For instance, primates have a remarkable aptitude at ranking each other within social hierarchies that are typically linear, and stable over long periods of time (Cheney and Seyfarth, 2007). The transitive inference (TI) paradigm has several attractive properties that lend itself to studying the mechanisms that support relational knowledge about hierarchies. For example, a capacity for social transitivity judgments is viewed to be supported by knowledge about the social hierarchy (Paz et al., 2004; Cheney and Seyfarth, 2007). Further, this knowledge is thought to develop through observation of pairwise interactions between conspecifics (Paz et al., 2004; Grosenick et al., 2007), mirroring learning during training trials in a TI paradigm. However, two important factors have previously limited the use of the TI paradigm in studying how knowledge about hierarchies develops and is represented: first, as discussed above only a minority of subjects develop hierarchy knowledge in the standard TI paradigm (reviewed in Zeithamova et al., 2012)—and secondly, that multiple mechanisms are known to contribute to successful transitive inferences in a typical TI paradigm (Frank et al., 2003; Zeithamova et al., 2012). As such, it has been difficult to specifically target the contribution of relational knowledge of the hierarchy to transitive inferences, over mechanisms based on retrieval-mediated associative linking (Kumaran and McClelland, 2012)and the use of elemental values (Fersen et al., 1991; Frank et al., 2003). The present study—by motivating a version of the TI paradigm that ensures robust hierarchy learning—provides an experimental setting, which lends itself to further characterization of the cognitive and neural mechanisms underlying hierarchy knowledge (see Kumaran et al., 2012 for a recent fMRI study using this approach).

## CONCLUSION

Taken together, our study has several implications: first, we establish the importance of task instructions in determining performance in the TI paradigm—and show that prior knowledge that item relations are transitive facilitates inference performance and the development of hierarchy knowledge, likely by constraining the space of possible task solutions. Second, our findings demonstrate that hierarchy recall accuracy, but not awareness scores, was a significant predictor of inferential performance across the entire group of participants—suggesting that this measure may be the more sensitive index of relational hierarchy knowledge. Finally, our instructional manipulation—combined with a specific measure of hierarchy recall accuracy—motivates an experimental setting which lends itself to further characterization of the cognitive and neural mechanisms underlying hierarchy knowledge.

**Figure A1:**
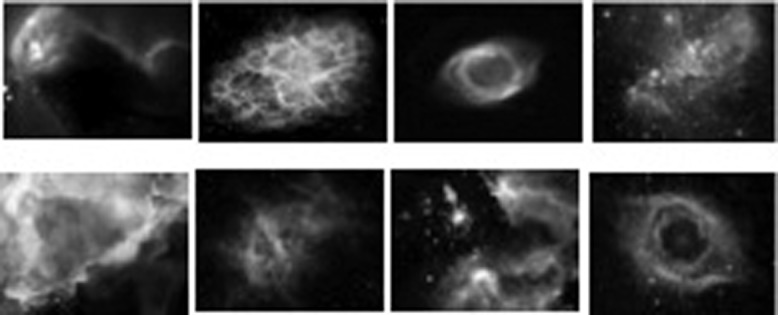
Example galaxy stimuli.

## APPENDIX

**Post-experimental “Awareness” Questionnaire** (based on Moses et al., 2006: scoring scheme (total score = 12) in italics within brackets, and not shown to participants)

1. How did you find the task? What strategies were you using?

(1a) Were all of the pairs in the testing sessions the same as in the training sessions?

YES (*0*)

NO (*2*)

NOT SURE (*1*)

(1b) Do you think there was a correct answer for the pairs in the testing condition?

YES (*2*)

NO (*0*)

NOT SURE (*1*)

(1c) If you believe there was a correct answer, explain why. (*A score of 2 was awarded to participants who described that there was a galaxy hierarchy. A score of 1 was awarded to participants who described that the relationship between items was transitive in nature, but did not describe a hierarchy. A score of 0 was given to other answers*.)

2. In the testing sessions (i.e., no-feedback) of the experiment you were presented with several different pairs of galaxies: what reason did you have for choosing one as opposed to the other? (Please tick one and explain why if possible)

- There is a logically correct choice. (*2*)
- One just seemed right but I can't explain why (*1*)
- I guessed: There may be a correct object, but I don't know what it is. (*1*)
- I made a random choice because there is no correct choice. (*0*)

3. What strategy (if any) did you use to learn which galaxy was correct? (Please tick one)

- I memorized each galaxy (*0*)
- I just watched and eventually got it (*1*)
- No strategy (*0*)
- I put them into an order/hierarchy (*2*)
- Other (scored as for question 1c)

4. Number the galaxies according to how you think they were ordered. Use the space provided to number each object 1 through 8. (*Galaxies were shown in random order: example shown. For the purposes of the questionnaire scoring, a score of 2 was given for perfect recall of the hierarchy order. A score of 1 was given if the position of 2 items was swapped, and 0 for more errors*.)

5. How confident are you of the order? (1–6) 1=guess, 6=very sure. (*This measure was not included in the final questionnaire score*).
